# Works on Pathological Anatomy

**Published:** 1834-10-01

**Authors:** 


### WORKS ON PATHOLOGICAL ANATOMY.

I. Principles and Illustrations of Morbid Anatomy, &c. &c. By *J. Hope*, M.D., F.R.S., Physician to the St. Mary-le-bone Infirmary, &c. Part XII. July, 1834.

II. Illustrations of the Elementary Forms of Disease. By *Robert Carswell*, M.D. Professor of Pathological Anatomy in the University of London, &c. &c. Fasciculus Sixth. Hæmorrhage.

The works before us are approaching their completion; the first, indeed, has reached it. The twelfth and last fasciculus of Dr. Hope's Illustrations of Morbid Anatomy, renders the whole a consistent treatise, displaying the lesions of the various organs, and presenting in a cheap and a portable form that kind and that amount of knowledge which is now indispensable for the surgeon and physician.

It would be idle to dwell on the benefits that have accrued, and must yet result from the due cultivation of that science which dissects disease—which unravels its processes and explains its history—which tells us what to dread and what to combat, when to act and when to stop—and which confers on medicine all that it can have of exactness and of certainty. We have said more than once upon former occasions, that *we* do not anticipate ultimate evil from the general study of morbid anatomy. In lieu of entertaining the opinion that that study has already gone too far, we are confident that it has not gone far enough. A little knowledge in science is a dangerous thing, because it leads to theory—a great deal lays the airy phantom that was raised, because it is productive of exactness. Look at the condition of medicine formerly, and regard it now. A century ago it was a thing of nostrums and tradition—aphorisms and precepts founded on empirical observation debased the mind and directed the hand of the practitioner of physic—and on more than one occasion, the progress of truth has been opposed by the buckler of ancient dogmatism and of consecrated error.* Now re-

* The introduction of lithotomy was long delayed, and its value when adoptmedies are directed against actual conditions—their actions on peculiar textures and on morbid processes are tested by experiment, and rated by experience—and the surgeon or physician wields against an enemy he knows, weapons which he understands. But we need not pursue the apology or the praise of morbid anatomy. It is enough for us, and for the practical portion of the argument, that the cultivation of it *must* proceed, and that those who aspire to possess even the current information of the day, must, willing or unwilling, be familiar with its facts.

The progress that has been made in this department is most gratifying. The work of Dr. Baillie was deemed a splendid effort, and no doubt at the time when it appeared it was so. Yet compare his brief notice of morbid alterations with the careful, laborious, and accurate investigations of the best pathologists of the present day. The advance has indeed been a giant's stride.

We hail the publication and diffusion of the works of Dr. Hope and Dr. Carswell, with feelings of deep satisfaction. They will carry the spirit of observation and research, and convey the results of labour and of thought to the surgeon of the distant and the quiet village, as well as to the busy hospital-physician. The former sees, with the assistance of lithography, diseases which had otherwise never met his eye; and the latter is stimulated to fresh enquiry and to more minute investigation, in order to confirm or to confute opinions. But we cannot permit ourselves to indulge any further in a strain of general remark, and we proceed at once to discuss the provender which fills our manger.

1. Dr. Hope's fasciculus is devoted to diseases of the brain and spinal cord. He remarks, with justice, that much as has been done in investigating the morbid anatomy of these organs, our knowledge is yet very far from satisfactory; symptoms occurring without definite corresponding lesions, and varieties of change of structure being unattended with similar varieties of symptoms. Dr. Hope has not depicted all the morbid alterations that are actually presented, for some, as induration and slight degrees of softening are incapable of just delineation. For reasons which we need not particularly notice, he confines himself to the description and the representation of the more important genera of disease, and treats in successive order of inflammation of the membranes and substance of the brain—softening of the latter—suppuration, abscess, and ulcer—induration—apoplexy—hypertrophy, atrophy, and anæmia of the brain and spinal chord—tubercle, scirrhous, encephaloid, fatty, fibrous, cartilaginous, and osseous productions, and hydatids. We must exercise our judgment in disposing of these various subjects. Dr. Hope's abridgement must be further abridged.

### Inflammation of the Membranes and Substance of the Brain.

Meningitis is the term which is applied to inflammation of the membranes in general: arachnitis is appropriated to that of the arachnoid: and sub-arachnoid meningitis implies inflammation of the sub-arachnoid cellular tissue and the pia mater.

"The dura mater is less frequently inflamed than the other membranes, and its external or fibrous, less frequently than its arachnoid layer. Its fibrous layer, in fact, is seldom inflamed, except from external injuries. In several cases of fracture of the skull, and in some of injury of the scalp alone, I have found pus,

---

ed, was at first debased, by the servile submission to the Hippocratic dogma, that membranous parts should not be cut.

either liquid or of pasty consistence, between the bone and the dura mater, and adhering to both. The bone was usually discoloured, and presented a zone of redness around the purulent deposition ; the dura mater exhibited a corresponding zone, and its arachnoid surface was vascular, had lost its polish, and appeared thickened. In such cases the disease is rarely more than a few inches in extent, corresponding with the external injury." 278.

We think that inflammation of the external surface of the dura mater is more frequent, and sometimes more extensive, after injuries of the head than Dr. Hope would appear to suppose. Most of us remember the numerous cases of suppuration on the dura mater detailed by Mr. Pott; and many have been surprised at his remarkable examples of success from the operation of trephining. Since the days of that able and eloquent surgeon, a better system of management and treatment have materially diminished the number of instances of suppuration on the dura mater. Yet still it is not an unfrequent consequence of scalp-wounds and of fractures of the cranium. We have seen such suppuration occupy the greater part of the surface of a hemisphere, and extend to near the basis of the skull. There is a circumstance connected with this kind of suppuration, which would seem to have escaped the notice of our author, but to which Sir B. Brodie has directed particular attention. It is this :—When suppuration has occurred *on* the dura mater, inflammation and suppuration generally occur, as a consequence, *under* it. Thus, a person receives a scalp-wound—suppuration ensues upon the bone, and follows, in succession, between it and the dura mater, and between the latter and the arachnoid covering the pia mater, or even beneath the arachnoid itself. A knowledge of this fact is essential to the surgeon.

Inflammation of the arachnoid layer of the dura mater is very uncommon. It is usually partial, but sometimes it is general, and the base is then the part last affected. The ramiform vascularity of its commencement may advance to uniform redness, and to effusion of serum, of lymph, or of pus.

In inflammation of the arachnoid, little or no vascularity is usually noticed after death, but sometimes its vessels are finely injected. The most constant change is, a degree of milky or opalescent opacity, attended, after a time, with slight thickening and increased consistence and tenacity, so that the membrane readily admits of being peeled off from the pia mater. The vertex and the base are the parts at which these changes are most common, the whole of the membrane being rarely affected. When the arachnoid is inflamed, the cellular tissue beneath it almost always participates in the affection. Serous effusion into the cavity of the arachnoid, and into the sub-arachnoid cellular membrane, and inflammatory injection of the pia mater, present no peculiarities that need detain us.

A frequent appearance is partial or general thickening of the arachnoid, and a milky cloudiness on the surface of the brain, partly occasioned by the thickening of the membrane, partly dependent on effusion of serum, or even of gelatinous lymph beneath it. Few bodies of persons advanced in life fail to exhibit more or less of these appearances. They are such as may, and frequently must arise from chronic inflammatory action. Yet we see them under circumstances so varied and so opposite, where cerebral symptoms have been present, and have not existed, that they seem to resemble the alterations in the coats of the arteries, and other changes incidental to old age, the inflammatory origin of which is problematical.

Obliteration of the sinuses of the dura mater is occasionally witnessed. It is supposed to arise from inflammation of those vessels. They are found choked up (and the great longitudinal sinus displays the fact in an eminent degree) with coagulum of blood, which strongly adheres to their parietes. In the centre of the coagulum is a semi-concrete puriform matter. Wherever the coagulum appears most recent, this central softening does not exist.

We have noticed this appearance on several occasions, and, however disposed to respect the authority which attributes its origin to inflammation, we doubt if that opinion is in all cases correct. Certainly we have observed this alteration in cases where no symptoms of phlebitis, nor even of material cerebral disturbance, were presented. It has seemed to us to occur in individuals who were long in dying, and we think that it resembles the polypi, with central softening, in the heart, which have also been remarked in cases where the final struggle was protracted.

However this may be, the consequences of obliteration of the sinuses of the dura mater are, not only stagnation of the blood in the cerebral veins, but exhalation of serum, and even of blood, into the cavity of the arachnoid, extensive ecchymoses in various parts, extravasations of blood in the sub-arachnoid cellular tissue, with softening of the cerebral tissue of the convolutions; rupture of vessels in the substance of the brain, and apoplectic coagula in the midst of the hemispheres. All these different effects result, without doubt, from differences in the seat and extent of the venous obstruction, and still more, perhaps, from the rapidity with which the sinuses are obliterated.

In describing inflammation of the substance of the brain, Dr. Hope successively passes in review its natural appearance—congestion—and genuine inflammation. To the former we need not allude, as words convey imperfectly a knowledge of those natural and standard features, which frequent opportunities of dissection can alone render familiar. Yet we may observe, that the vascularity of infancy and early youth confers upon the brain a pinkish hue, while the weakened circulation of old age is productive of a yellowish tint. But the diagnosis of congestion and of inflammation is important, and induces us to notice the statements of our author. And, first of cerebral congestion.

This is augmented by diseases attended with obstruction to the return of the blood from the head, such as asthma—asphyxia—convulsions, &c. Hypertrophy of the left ventricle of the heart occasions it, by producing a preternatural determination to the brain. If the head is placed in a dependent position after death, gravitation of the blood may occasion very considerable injection, and even uniform redness; and the latter may occur, if dissection is delayed till putrefaction has commenced. Cerebral congestion is diminished by opening the chest before the head, the blood then draining off by the jugular veins. Exposure of the stripped surface or sections of the brain to the air is well known to augment its redness, by the influence of the atmosphere upon the blood. We subjoin Dr. Hope's description of congestion.

"The veins on the surface are preternaturally turgid, and their minute ramifications become unusually distinct. The interior exhibits an exaggerated degree of the natural red dotting, especially in the grey substance of the convolutions, the colour not being so bright a red as that from inflammation. In cases of great and old congestion, it is not uncommon for the vessels of the medullary substance to be so charged with dark blood as to impart a uniform dingy grey tinge to this substance. Dr. Bright has shewn that the vessels on which this colour depends are easily perceptible with a lens. In some instances, more especially those in which the circulation has been greatly retarded, the brain is marbled with a purple cloudiness, occasioned, perhaps, by a faint transudation through the gorged vessels. When the obstruction to the return of the blood from the brain has been extreme, as from obliteration of the sinuses by coagula, rupture of the minute vessels of the cerebral substance sometimes occurs and forms numerous petechial extravasations, which, when very close to each other, break down the texture of the part. They are most abundant in and near the grey substance of the convolutions. Similar extravasations are also very common in the vicinity of apoplectic effusions; being sometimes the result of violence done to the surrounding parts by the clot, and sometimes a product of the original tendency which led to the apoplectic effusion. Concussion of the brain often causes the same ecchymoses by laceration of the minute vessels.

Congestive redness may not only be dotted but uniform. This variety of redness, indeed, is most commonly a result of congestion, and is much less frequently the anatomical sign of inflammation than speckled redness. (Andral.) Uniform redness may vary in depth, from a light rose-colour to a deep mahogany hue. It is never general, being found only in patches in different parts. It occurs in either of the two substances, but principally in the grey, both of the convolutions and of the *corpora striata* and *thalami optici*. In the white substance, where it is rare, it is usually found in the vicinity of apoplectic effusions, though it may occur independent of hæmorrhage." 285.

In the brain, as in other organs, the blood, whether extravasated or contained within its vessels, may, from various causes, pass through various tints.

The foregoing characters of congestion may now be contrasted with those of inflammation. No risk of mistake exists on the part of the experienced anatomist. But all our readers have not great opportunities of pathological investigation, and, therefore, we dwell the more strongly on the subject. Serious errors have undoubtedly ensued, from an imperfect acquaintance with the respective morbid states.

"In inflammation (proceeds Dr. Hope), the substance of the brain, when sliced, presents a preternatural degree of scarlet dotting, which has been compared by M. Lallemand to a white surface sprinkled over with red sand (*'injection sablée.)* The dots are occasioned by blood circulating in the naturally colourless capillaries, from the larger of which it may be seen oozing for some seconds after the incision has been made. In young children, the injection is occasionally so great as to give a uniform pink tinge to the whole brain. Intermixed with the dots, are small red stains, probably occasioned by rupture of the capillaries and infiltration of blood into the surrounding pulp. These stains are various in number, size, form, and depth of colour: sometimes they give a marbled aspect to the part; and sometimes, by coalition, they form patches of red, the depth of which decreases from the centre, towards the circumference of the patch. These marks of inflammation may exist either in scattered parts of the brain, or may affect a considerable portion of a hemisphere. The vascular turgescence imparts an increased degree of firmness to the part inflamed; but, at the same time, it is more lacerable than natural. The diagnosis of inflammatory from congestive redness will be facilitated by retaining in recollection that the former, when recent, is of a more brilliant or scarlet hue; that it is usually accompanied with anatomical vestiges of inflammation of the membranes, and with the symptoms of cerebral inflammation. When cerebritis is extensive, it may prove fatal without advancing beyond the degree now described, especially if accompanied by meningitis; when limited, it commonly passes into the second degree, or that of softening." 286.

Inflammation of the cerebrum is usually attended with an effusion into the ventricles of serous, or more rarely, of sero-purulent fluid, the quantity of which is never great when the inflammation is acute; in chronic inflammation it may be enormous. Cerebritis is most frequent in children.

### Softening of the Brain.

On this we will not enter, as the lesion was described in the last fasciculus of Dr. Carswell, and has been already noticed in this Journal.*

### Suppuration, Abscess, and Ulcer of the Brain.

On suppuration and abscess Dr. Hope says little. All that we need notice is the passage which refers to simple ulceration of the brain, an alteration which we have heard a most experienced surgeon deny having ever observed. This

* *See* the review of the fourth fasciculus of Dr. Carswell's Illustrations of the Elementary Forms of Disease. No. 41, for July, 1834—pp. 46-8.—Eds.

form of ulceration, says Dr. Hope, is rare. It affects the surface of the convolutions, and of the *thalami optici* and *corpora striata,* the nervous substance of which is superficially eroded, so as to form ulcers of various sizes and forms, mostly with uneven, ragged edges and a yellow albuminous surface, but sometimes hard and dry. The surrounding cerebral substance may be either healthy or injected. Occasionally, the ulcer communicates with deep-seated abscesses. Sometimes it originates in the arachnoid and pia mater. This species of ulcer is mentioned by various writers, as Morgagni, Scoutetten, and in the *Archives de Médecine.*

### Induration of the Brain.

Three degrees of this alteration have been described. The first is of the consistence of brain that has been immersed for some time in diluted nitric acid—the second equals the firmness of wax—and in the third, the brain is as firm and elastic as fibro-cartilage.

Induration may be either general or partial. When general, it has never been observed to exceed the first degree. The medullary substance may contain little or no blood, and is of remarkable whiteness : it is firmer than the grey, especially in the central parts of the brain and at the origin of the nerves. General induration has been observed principally after ataxic fever, and after convulsions from the poison of lead. It appears to be usually an acute affection. Induration may affect the whole length of the spinal cord, being here also principally confined to the white substance.

Partial is more common than general induration, and is usually a result of chronic inflammation. In many cases, however, the existence of inflammatory action is only a matter of dubious inference. Partial induration may exhibit various tints of red or yellow ; or it may possess the colour as well as the density of gristle. The disease has been observed in different parts of the nervous centres, and it sometimes affects the grey substance of the convolutions exclusively. After subsisting for a considerable time, it occasionally terminates in suppuration.

### Apoplexy.

So much has been written and said upon this disease, that our memoranda will be very brief. One or two insulated observations will complete them.

Apoplectic extravasations of blood are much more rare on the surface and within the ventricles, than in the substance of the brain. Andral mentions that, of 392 cases of cerebral hæmorrhage which he has found described by authors, the seat of it was in some part of the substance of the brain in so many as 386 ; in 202 of which it occurred in the part of the cerebral hemispheres on a level with the *corpora striata* and *thalami optici,* as well as in those parts themselves. Our limited observation would induce us to believe that extravasation of blood into the ventricles of the brain is more frequent in comparison with extravasation in its substance, than the ratio of 1 to 65.* Yet M. Andral's authority and calculations are equally entitled to respect.

Dr. Hope describes from successive preparations the series of changes displayed by apoplectic extravasations, from the first effusion, to their final absorption and the formation of a cicatrix. Moderate apoplectic extravasations are curable, but one is usually followed by others, for, unfortunately, the original cause remains. Several months appear necessary for the completion of a cicatrix.

---

* Yet probably we are incorrect; for M. Andral limits his instances of effusion into the cavity of the ventricles, to such as are unaccompanied by any laceration of the substance of the cerebrum. The extravasation must, in fact, be furnished by the vessels of the ventricle.—*Eds.*

Dr. Hope properly and forcibly draws attention to the connexion between sanguineous apoplexy and organic disease of the heart. It is indeed a connexion with which, on all accounts, the practical surgeon or physician should be well acquainted. Did we trust our own experience and our own observation, we should say that this form of apoplexy seldom happens unless the arterial system be diseased, and that the latter rarely is so unless there be hypertrophy of the left side of the heart. There are exceptions, it is true, to this general rule, but as a rule it is usually correct, and a wide expression of this description is of immense importance to the practical man. Indeed, we should say that, in practice, the great value of morbid anatomy is the generalizations which it permits the scientific practitioner to make, generalizations which assist him in individual cases in the hour of need.

### HYPERTROPHY, ATROPHY, AND ANÆMIA OF THE BRAIN AND SPINAL CORD.

On these alterations our author is brief, and we shall be silent.

### TUBERCLES, SCIRRHOUS, ENCEPHALOID, FATTY, FIBROUS, CARTILAGINOUS, AND OSSEOUS PRODUCTIONS, AND HYDATIDS.

Tubercle and fatty alteration are all that need detain us. The latter is so easily dispatched, that we may begin with its consideration.

A large mass, like adipocire, was found by Dr. Leprestre; and one as large as a hen's egg, and resembling spermaceti, was met with by M. Dalmas. The latter consisted of a large quantity of fatty matter, and another matter seeming to be cholesterine.

Tubercles of the brain and spinal marrow are principally found in children, being rare in infants, and still more so in adults. They are few in number, and sometimes there is only one. Their colour is commonly a pale greenish-yellow, but, when the centre softens, it becomes of an ochre-yellow. Their consistence is somewhat less than that of pulmonary tubercles; and, like the latter, they sometimes acquire a cretaceous composition, in colour and consistence resembling dryish putty. Their most common size is about that of peas, but they may be as small as millet-seeds, or may even exceed the size of hen's eggs; when grouped, they may form still more considerable masses. They would seem to be most commonly generated in the pia mater; and when they arise in the cerebral substance, it is usually in or near the grey matter. The parts where they are found, next in order of frequency to the hemispheres, are, successively, the cerebellum, the pons varolii, the medulla oblongata, various parts of the spinal cord, (especially about the cervical region,) the crura cerebri, the crura cerebelli, the thalami optici, the corpora striata, and the pituitary gland. Tubercles are often surrounded by a cyst, which is generally fine, but sometimes thick, and even fibrous, cartilaginous, or osseous.

Here we close our notice of this Fasciculus, and, with it, of the work of which it forms a portion. The whole does no discredit to its author, a gentleman now honourably distinguished by his talents, assiduity, and zeal. We wish him the success which he deserves and will obtain. For the work itself we will only say that its form, its value, and its cheapness conspire to render it an acquisition to all who would keep pace with the pathological knowledge of the day. The country-surgeon or physician would find himself repaid in its purchase and its study.

---

II. We now turn to the sixth Fasciculus of the work of Dr. Carswell. It is dedicated to the description and the delineation of hæmorrhage in the various textures in which it occurs.

Hæmorrhage, in the proper acceptation of the term, implies the escape of blood from its vessels, into the substance or on the surface of organs, whether it be retained in these situations, or conveyed to the exterior of the body. It may take place from any portion of the vascular system, in consequence of a solution of continuity by wound, ulceration, &c.; or it may proceed from the capillary vessels without any obvious lesion. Hæmorrhage may result, too, from morbid alterations of structure or from morbid growths, but in them it must occur in one of the two above-mentioned modes.

Various circumstances operate in producing or in giving a tendency to hæmorrhage. Diseases of the heart and of the vascular system are, perhaps, the most efficient of such causes. The alterations of the heart consist principally of hypertrophy, with or without dilatation, and "of the non-occlusion of the auriculo-ventricular orifices, especially on the left side of the organ, in consequence of permanent retraction of their valves." The result of these morbid states, opposing as they do the return of the venous blood, is venous congestion, which increasing more in some organs than in others, terminates in rupture of the capillary vessels, or in exhalation of blood from their interior. Dr. Carswell has not noticed a frequent cause of extravasation of blood into the lungs:—we allude to contraction of the left auriculo-ventricular aperture. We published several cases of this nature in a previous number of this journal. The rationale of such hæmorrhage is too obvious to require comment. We do not perceive, so clearly as Dr. Carswell, the necessity for congestion in the venous system in cases of hypertrophy of the left side of the heart. Extravasation of blood into the brain is a frequent effect of that disease, but it appears more natural to attribute the rupture of the cerebral vessels to the great and unnatural impulse of the ventricle, than to suppose that this, which is morbidly strong, should be unequal to propel the blood through the veins.

The condition of the blood-vessels is extremely operative in the production of hæmorrhage. The deposition of calcareous and ossific matter in the coats of the arteries—softening of those coats—and loss of elasticity of their lining membrane, are morbid alterations which are known to attend and to occasion apoplexy, aneurism, and various extravasations. It is worthy of remark, that the small vessels of the brain are very liable to rupture, and are seldom aneurismal, while the converse is the case with the larger arteries of the body. The absence of a cellular sheath to the former vessels appears to furnish the explanation of the fact.

Local congestion in the dependent parts of the bodies of those advanced in age or debilitated by disease—and in the substance of various organs, unattended by any lesion excepting extreme fluidity of the blood, are circumstances that precede or accompany hæmorrhage. Vicarious congestions are also not unfrequent, and give rise to vicarious hæmorrhages. Such congestions are boldly attributed by Dr. Carswell to a modification of a function of the capillaries, an explanation which, when analysed, is little more than a learned periphrasis for our ignorance upon the subject.

Prior to describing the physical character of hæmorrhage in the organs in which it most frequently occurs, Dr. Carswell presents a general view of its locality, its local consequences, and the more important changes which occur in the effused fluid. On these heads our notice will be brief.

The locality of hæmorrhage is extremely varied. Several of its forms are only observed in particular organs; others are not restricted in this manner, although they are more frequent in some organs than in others. Age is well known to exert an important influence. Hæmorrhage from the nose is most frequent in childhood—from the air-cells and bronchi in youth—from the digestive, uterine, and urinary organs in middle age and the decline of life—in old age from the brain. Mucous membrane is more liable to hæmorrhage than any other elementary tissue.

The observations on the *seat* of hæmorrhage are deserving of attention. Yet the principal interest is connected with the situation of apoplectic extravasations, and our notice of the last Fasciculus of Dr. Hope precludes the necessity of our reverting to the subject. The *quantity* of blood effused is various, and arteries usually furnish more than veins. In a case in which death ensued in ten minutes from an ulcerated varyx, Dr. Carswell found the walls of the vein so thickened, and so firmly united with indurated cellular tissue, that considerable pressure was necessary to approximate their internal surface. It was impossible for contraction of the aperture to occur.

The local effects of hæmorrhage are compression, laceration, obstruction of natural passages, inflammation, suppuration, and mortification.

On the consequences of compression we shall say no more than is sufficiently expressed in a general law laid down by Dr. Carswell:—that the disturbance of the functions of an organ from pressure is in the direct ratio of the rapidity with which the compressing cause operates. On the other local effects of hæmorrhage it is not necessary to dwell.

The changes which take place in the effused blood occupy at some length the attention of Dr. Carswell. We cannot follow him, and perhaps it will be sufficient to observe, that effused blood is either removed, or remains and becomes organized—that the removal by absorption is effected with more readiness in some tissues than in others—and that its organization is marked by the successive phases of coagulation, vascularization of the fibrine, and gradual absorption of the latter, giving rise to the appearance of a cicatrix. Sometimes, instead of a cicatrix of this description, the organised fibrous substance is converted into a loose cellular tissue, filled with a serous fluid, and generally traversed by a considerable number of blood-vessels. As the quantity of the serous fluid increases that of the cellular tissue and the vascularity diminish, and thus a considerable cavity is formed, filled with yellow serum, and bounded by the remaining cellular tissue, which at length is changed into a cyst. The obliteration of this is the next circumstance remarked. It is accomplished by the gradual removal of the fluid, and the approximation of its walls, which become united, and form a cicatrix. In some few cases the latter has been found to disappear, the only morbid appearance remaining being a change in the bulk and direction of the grey and white fibrous structure of one of the thalami or corpora striata, in which we must suppose the extravasation to have originally been.

The physical characters of hæmorrhage are next described by Dr. Carswell. He delineates them successively in the brain, the lungs, the digestive, urinary, and generative organs, and finally, in the skin and the cellular texture. The physical characters of effused blood are almost obvious, and certainly simple. We shall scarcely touch upon them.

Of hæmorrhage in the brain we need say no more.

In the lungs, hæmorrhage presents three varieties:—in the first, the blood is contained within the vesicular structure of the lung, and forms a round, circumscribed, solid mass, varying from half an inch to two inches in diameter; in the second, the blood is effused into the cellular tissue, in which it spreads extensively and rapidly, forming a ragged excavation, occupied with blood and portions of engorged and torn cellular tissue; the third variety is a consequence of the second, and consists of the super-addition of rupture of the pleura.

Of hæmorrhage from the digestive organs we will only say, that its black colour is due to the action of an acid on the blood. Follicular ulceration is the most frequent lesion attendant on gastric and intestinal hæmorrhage.

Hæmorrhage from the urinary organs seldom attains considerable magnitude, unless there be malignant disease of the bladder. The medullary fungus is the usual form.

Hæmorrhage from the uterus may be occasioned by congestion of its mucous membrane—by ulceration of the os tincæ or vagina—by carcinoma in either situation—by polypi within the cavity of the uterus. The ovaries are sometimes the seat of hæmorrhage which distends their capsule.

Hæmorrhage of the skin and cellular tissue constitute petechiæ, purpura, and scurvy. On these affections Dr. Carswell offers nothing new.

Of the plates of this fasciculus we can only make one remark—they are beautiful.

